# Nanocomposite PLA/C20A Nanoclay by Ultrasound-Assisted Melt Extrusion for Adsorption of Uremic Toxins and Methylene Blue Dye

**DOI:** 10.3390/nano11102477

**Published:** 2021-09-23

**Authors:** M. Andrade-Guel, C. Cabello-Alvarado, R. L. Romero-Huitzil, O. S. Rodríguez-Fernández, C. A. Ávila-Orta, G. Cadenas-Pliego, D. I. Medellín-Banda, C. Gallardo-Vega, J. Cepeda-Garza

**Affiliations:** 1 Centro de Investigación en Química Aplicada (CIQA), Saltillo 25294, Mexico; carlos.avila@ciqa.edu.mx (C.A.Á.-O.); gregorio.cadenas@ciqa.edu.mx (G.C.-P.); diana.medellin@ciqa.edu.mx (D.I.M.-B.); carlos.gallardo@ciqa.edu.mx (C.G.-V.); jesus.cepeda@ciqa.edu.mx (J.C.-G.); 2 CONACYT—Centro de Investigación y de Innovación del Estado de Tlaxcala, Tlaxcala 90000, Mexico; 3 Facultad de Ingeniería Química, Benemérita Universidad Autónoma de Puebla, Puebla 72000, Mexico; ruth_lissette@hotimail.com

**Keywords:** nanoclay, poly (lactic acid), uremic toxins, methylene blue, adsorption

## Abstract

**Design of functional materials** it is of great importance to address important problems in the areas of health and environment. In the present work, the synthesis and application of poly-meric nanocomposite materials with poly (lactic acid) (PLA) and modified nanoclay (cloisite 20A) with 1,4-diaminobutane dihydrochloride at different reaction times were studied. The concentra-tions of the nanoclays in the PLA matrix were 0.5, 1 and, 5% by weight (wt%). TGA showed that sample C20AM 120 (120 min of treatment) obtained the highest degree of modification considering the weight losses of the analyzed samples. An FT-IR signal at 1443 cm^−1^ suggests that the organic modifier is intercalated between the galleries of the clay. XRD, SEM and XPS suggest good disper-sion at low concentrations of the nanoclay. Adsorption tests revealed that the highest percentage of removal of uremic toxins and methylene blue was the sample with 5% wt/wt chemically modified nanoclay, suggesting good affinity between the modified nanoclays in the PLA matrix with the nitrogenous compounds.

## 1. Introduction

There are pollutants that are very harmful to humans, both in the blood (uremic toxins) and in water (methylene blue) that contain nitrogen groups; these pollutants directly or indirectly affect the health of people and their environment. Chronic kidney disease is a progressive loss of kidney function caused by the accumulation of toxins in the blood [1]. The contamination of waters, rivers, and lakes seriously affects the whole world. Approximately 200,000 tons of synthetic dyes-contaminated water are discharged into effluents annually [2,3,4]. 

Filters or membranes based on non-biodegradable polymers are used to mitigate some of these problems, which act adequately in filtration processes. A wide variety of membranes are prepared based on synthetic polymers, which take thousands of years to degrade generating contamination of different kinds. Besides, sometimes their reuse is limited. For these reason, in recent years different biodegradable polymers have been studied, to be used as media filters and applied at an industrial level depending on the manufacturing process [5,6,7].

Polymers such as polyurethane, polyacrylonitrile, polyvinylidene fluoride, polyvinyl chloride (PVC)/PU, polycarbonate, silk, polyimide and PLA poly (lactic acid) [4] have been used in the production of air filtration media, the latter having good biodegradable properties. Lactic acid is a precursor of poly (lactic acid) (PLA), with biodegradable characteristics [8]. The production of 90% of the LA (lactic acid) in the global market is by lactic acid bacteria, which can selectively produce stereo L or D-LA. The remaining portion of LA produced is synthesized by lacto nitrile hydrolysis, base-catalyzed degradation of sugars, propylene glycol oxidation, etc., resulting in the racemic mixture of LA enantiomers [9]. There is the possibility of obtaining nanocomposites with different polymers, and nanoreinforcements as additives. Biodegradable polymer nanocomposites have been of great interest and impact to society, since there is great concern about the growing environmental pollution of synthetic polymers and the large amounts of waste that are housed in different aquifers, damaging their vegetation and animals [10,11,12]. Nanoclays, due to their structure and composition, are used as nanoreinforcements, because they show a high aspect ratio and a large surface area [13]. This type of nanoparticles is composed of phyllosilicates, and in some studies, they are used as adsorbents for organic compounds, mainly due to their large surface area, high cation exchange capacity, and relative ease of forming complexes of intermediate layers. Considering the above advantages, the nanoclays are a favorable candidate for the reinforcement of polymeric materials with possible use as materials to fabricate membranes and/or adsorbing filter contaminants [14]. 

Iturrondobeitia et al. studied the thermal stability of PLA/Cloisite 20A and Cloisite 30B nanocomposites. These authors observed the changes that nanocomposites presented when modifying extrusion, and injection molding processing parameters [15]. Several studies have been carried out on the preparation of nanocomposites based on PLA with cloisite for application as a water vapor barrier, antimicrobial properties [16], thermal properties [17] and mechanical properties [18,19,20].

Some of the changes that occur in the nanocomposite can be attributed to an interfacial interaction between the polymer matrix and the chemically modified layers of silicates, which helps this interaction [21,22,23]. Among the different techniques for obtaining nanocomposites, the melt extrusion method is considered flexible, economical, compatible with industrial processes, and of high volume compared to the other existing techniques of polymerization in-situ and dispersion in solution [24].

The application of ultrasound in the melt extrusion process helps to disrupt the converging flow of the melt, and changing the flow patterns, which leads to a lower elastic tensile deformation. It also improves the movement of the polymer chains, so the elastic tensile deformations can be recovered very quickly, facilitating the dispersion of the nanoparticles in the polymer matrix [25,26,27]. Moreover, dispersion of nanoparticles is significantly improved with the application of ultrasound during melt extrusion [28,29].

Based on the above, the aim of this study is to determine the effect of nitrogenous compounds in variable frequency ultrasound-assisted chemically modified nanoclay (in this case 1,4-diaminobutane dihydrochloride) integrated to PLA in the adsorption of uremic toxins and methylene blue, and the possibility to use this designed material to produce a new filter medium in health and environmental areas. For this purpose, to ensure nanoparticle dispersion, ultrasound-assisted melt extrusion was used for the fabrication of poly (lactic acid) (PLA) and nanoclay Closite 20A (0.5, 1, 5, and 10 wt%) polymer nanocomposites.

## 2. Materials and Methods

### 2.1. Materials

Organoclay Closite C20A BYK with a particle size: <10 µm and lamellar spacing of 2.7 nm was used as starting material. As a clay modifying agent, 1,4-diaminobutane dihydrochloride with 99% purity (Sigma Aldrich, Saint Louis, MO, USA) was applied. Distilled water with a pH of 7 was used as a solvent to obtain the concentrated aqueous solution. Poly (lactic acid) resin Ingeo biopolymer 6260D was purchased from NatureWorks with a melt index of 65 g/10 min. Additionally, for the removal of toxins, Urea from Faga Lab was acquired. Creatinine anhydrates ≥98% purity from Sigma Aldrich, crystalline uric acid with purity ≥99%, Sigma Aldrich, and tests with Methyl blue (MB) were performed as a colorant. From Sigma Aldrich (Saint Louis, MO, USA), classification Acute Tox. 4; H302. 

### 2.2. Methods

#### 2.2.1. Chemical Modification of Cloisite 20A by Ultrasonic Tip

The chemical modification treatment was carried out by dispersing 1 g of C20A nanoclay in 20 mL of distilled water, with 1,4-diaminobutane dihydrochloride; in a 1:1 ratio, using a homemade ultrasonic generator, with an output power of 750 W, at an amplitude of 50% and a variable frequency of 15 to 50 KHz, catenoidal ultrasonic tip (Branson Ultrasonics Corp., Brookfield, CT, USA; D, 51.27 cm). For safety reasons, all experiments were carried out in a soundproof cage. Six different treatment times of 15, 30, 45, 60, and 120 min were used. All the experiments were carried out at room temperature. At the end of the experiments, the C20A clay was filtered and dried at 80 °C for 24 h.

#### 2.2.2. Composite Preparation by an Ultrasound-Assisted Melt Extrusion Process

PLA/nanoclay polymer nanocomposites preparation was carried out using the ultrasound-assisted melt extrusion process (US) to homogenize the mixture of the particles within the polymer. For the extrusion process, a lab-size twin-screw extruder from Thermo Scientific (model, Prism TSE-24MC) with a screw diameter of 24 mm and L/D ratio of 40:1 was assisted by a catenoidal ultrasonic tip (Branson Ultrasonics Corp., CT; D, 51.27 cm). The extruder was connected to a homemade ultrasonic generator (15 to 50 kHz, 100% of 750 W). This technique has been previously reported by the authors because it has been observed to favor the dispersion of nanoparticles [28,29].

**Figure 1:**
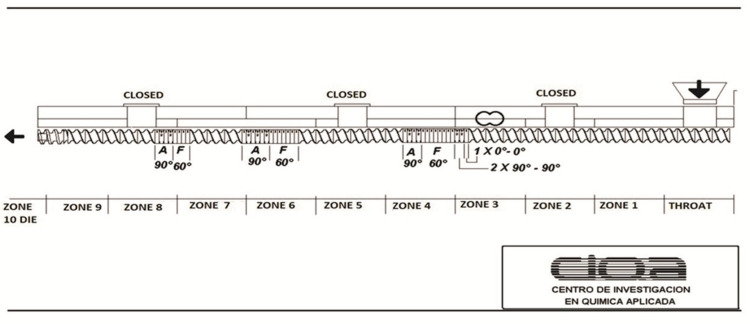
Screw mixing setup used during extrusion.

A low shear stress extrusion configuration was used to improve particle dispersion in the polymeric matrix (Figure 1). The temperature profile was a flat one, at 180 °C in all areas of the extruder, with a screw speed of 100 rpm [30,31,32]. These experimental conditions help the further homogeneous size of pellet. As a post-extrusion system, a cooling bath was used at the outlet of the die and a pelletizer (Thermo Fisher, Waltham, MA, USA). 

Table 1 shows modified clay samples according to the treatment time. The clay with 120 min of modification was chosen because it showed a better percentage of modification. Modified clay was identified as C20AM in the polymeric nanocomposites and adsorption tests. The formulations of each of the poly (lactic acid) and cloiside 20A nanocomposites that were prepared by ultrasound-assisted extrusion are listed in Table 2.

### 2.3. Characterization

#### 2.3.1. Fourier Transform Infrared Spectroscopy (FTIR) 

Fourier transform infrared spectroscopy (FT–IR) analysis was performed using a Nicolet Magna 550 FT-IR spectrophotometer. The conditions were 100 scans and a resolution of 16 cm^−1^ in the range of 400 to 4000 cm^−1^. Potassium bromide (KBr, Sigma Aldrich, Toluca, Mexico) powder is used to prepare KBr pellets for infrared analysis. The powder samples were previously dried in a vacuum oven at 80 °C for 24 h and then ground in a mortar.

#### 2.3.2. X-ray Diffraction (XRD) 

Structural analysis data obtained by performing XRD (X-ray diffraction) were collected on a Rigaku Smartlab diffractometer operating at 40 kV and 40 mA with stability of 0.01%/8 h. Measurements of each system were performed in the 2θ range from 10° to 80° with a step size of 0.02° and a counting rate of 10 s/step.

#### 2.3.3. Thermogravimetric Analysis (TGA)

To evaluate the thermal stability of compounds, thermogravimetric equipment (TA Instruments Q550, New Castle, DE, USA) was used. The samples were subjected to heating at a constant speed of 10 °C/min from room temperature to 700 °C under a constant nitrogen flow of 50 mL/min, subsequently, the temperature was raised at the same speed to 800 °C under an atmosphere of oxygen.

#### 2.3.4. X-ray Photoelectron Spectroscopy

The XPS study was realized in the K-ALPHA spectrophotometer (Thermo Fisher, Waltham, MA, USA) (Thermo Scientific, Waltham, MA, USA, model XL-30 Phillips instrument with an accelerating voltage of 5–25 keV) with a monochromatic X-ray source with a binding energy of 0–1350 eV and a depth of 400 µm, there is no pre-treatment for the samples.

#### 2.3.5. Scanning Electron Microscopy (SEM) 

For the determination of the dispersion and morphology of the nanocomponents, a field emission scanning electron microscope JOEL model JSM-7401F (JEOL, Peabody, MA, USA) was used. The acceleration voltage of the microscope was 3.0 KV using the LEI secondary electron detector. Pieces of compression cast plaques were cryo-fractured and coated with gold for SEM. The SEM observations were realized directly on the external and cryo-fractured surfaces of the composites.

#### 2.3.6. Transmission Electron Microscopy (TEM)

The morphology and elemental analysis of the unmodified, and 1,4-diaminobutane dihydrochloride-modified nanoclay was studied with transmission electron microscopy TEM (FEI-TITAN 80–300 kV) with an objective lens (Type S-TWIN; Cs = 1.3 mm).

#### 2.3.7. Adsorption Uremic Toxins

For the determination of urea, creatinine, and uric acid adsorption on C20A clay and PLA/C20AM US nanocomposites, three solutions were prepared with a 160 mg/L concentration of urea, uric acid, and creatinine. The adsorption experiments were carried out each in separate beakers 250 mL with 20 mL of the solution either urea, uric acid, or creatinine prepared above and 0.02 g C20A and nanocomposites PLA/C20AM US, respectively. The beakers were placed on a shaking rack at room temperature and vigorous shaking of 500 rpm for 4 h because it is the time of the hemodialysis treatment. Every 15 min a sample was taken and placed in a vial for later reading. All experiments were performed in triplicate. The removal percentage of each toxin was calculated with the following equation. Where *C_i_* is the initial concentration, *C_e_* is the final concentration.

$$\%\;Removal=\frac{\left(C_{i}-C_{e}\right)}{C_{i}}\times 100$$

For the analysis of uremic toxin adsorption, the samples were analyzed in a UV-VIS spectrometer (Shimadzu UV-2401 PC) at different wavelengths 200 nm (urea), 230 nm (creatinine), and 293 nm (uric acid). For the calculations of the Langmuir and Freundlich isotherms, the methodology reported by Cabello et al. was followed [33].

#### 2.3.8. Adsorption of Methylene Blue

For the determination of methylene blue adsorption on C20A clay and PLA/C20AM US nanocomposites, an aqueous solution with a concentration of 200 mg/L of methylene blue was prepared. The adsorption experiments were carried out in 50 mL beakers with 20 mL of the solution and 0.02 g of C20A and PLA/C20AM US nanocomposites, respectively. The beakers were placed on a stirring grill at room temperature and vigorously shaken at 500 rpm for 60 min. Every 15 min a sample was taken and placed in a vial for later reading in the UV-VIS spectrometer (Shimadzu UV-2401 PC) at a wavelength of 664 nm. All experiments were done in triplicate. 

The percentage of adsorption efficiency was calculated according to Equation (1):

(1) $$\%\;Adsorption\;efficienty=\frac{C_{i}-C_{e}}{C_{i}}\times 100$$

where *C_i_* and *C_e_* are initial and final concentrations, respectively.

The adsorption capacity of the nanoclay and PLA/C20AM was calculated with Equation (2) in equilibrium:

(2) $$q_{e}=\frac{\left(C_{i}-C_{e}\right)V}{m}$$

where *V* is the volume in L of solution and *m* is the amount of mass in mg of absorbent.

#### 2.3.9. Desorption Studies 

The desorption and regeneration of the adsorbents for uremic toxins and methylene blue were studied for four successive cycles. In each cycle, the adsorbents were loaded with uremic toxins or methylene blue by mixing 20 mg of the sample with 100 mL of the 160 mg/L concentration of urea, uric acid and creatinine or 200 mg/L concentration of dye at room temperature. The mixture was shaken for 1 h. The samples loaded with uremic toxins or methylene blue were separated by filtration and then mixed several times with distilled water. The percentage remove of the regenerated materials was determined by the same method mentioned above.

## 3. Results and Discussion

### 3.1. C20A Nanoclay Modification

#### 3.1.1. Thermogravimetric Analysis (TGA)

**Figure 2:**
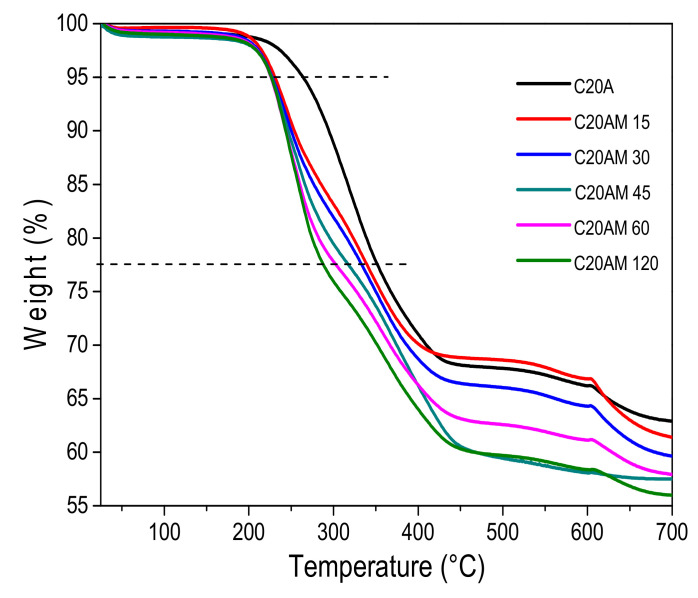
Termogravimetric analysis of the nanoclay C20A and modified nanoclay C20A with 1,4-diaminobutane dihydrochloride.

Figure 2 shows the TGA curves for the modified nanoclays, where it can be observed that the materials have different thermal behavior than the pristine nanoclay. This is due to the modifying agent and the time of ultrasound treatment that is given to each sample. The decomposition initial temperature has been reported at 198 °C for the C20A nanoclay [34], in this study, unmodified nanoclay begins its decomposition at 210 °C, while the modified one presented a decomposition temperature of 196 to 198 °C. Three weight losses were detected in all samples. In the case of the unmodified nanoclay, the first weight loss occurred from 210 °C to 423 °C with a loss of 30% in weight, this is due to the thermal degradation of the nanoclay. In the modified nanoclay samples the first loss was observed between 196 at 448 °C. For the sample C20AM 120 there is a weight loss of 40%, which was related to the presence of organic compounds on the surface of the nanoclay. The second weight loss ranges from 417 °C to 605 °C with a 2% loss in weight. In all samples, a shoulder at higher temperatures at about 605 °C is observed; based on previous work on the thermal degradation of organically-modified clays, this may be attributed to the release of olefin and amine [35]. Based on the thermogram, sample C20AM 120 is the one with the highest degree of chemical modification.

Table 3 presents the results of the mass loss temperatures at 5% (T_5%_) and at 25% (T_25%_) for the modification of the clay. The modified C20A samples showed weight loss related to the presence of organic functional groups on the surface of the nanoclay, this is attributed to a possible link between the silanol groups of the clay and the amino and chlorine groups of the modifier (1,4-diaminobutane dihydrochloride).

Ojijo et al. studied nano-biocomposites based on poly (butylene succinate) PBS and poly ((butylene succinate)-co-adipate) PBSA and clay. In the thermogravimetric analysis of the clays, they attributed the weight losses at temperatures of 200–350 °C to a possible chemical covalent bond between the silanol groups of the clay and the hydroxyl groups of the modifier (urethane) [36].

#### 3.1.2. Fourier Transform Infrared Spectroscopy (FTIR) 

**Figure 3:**
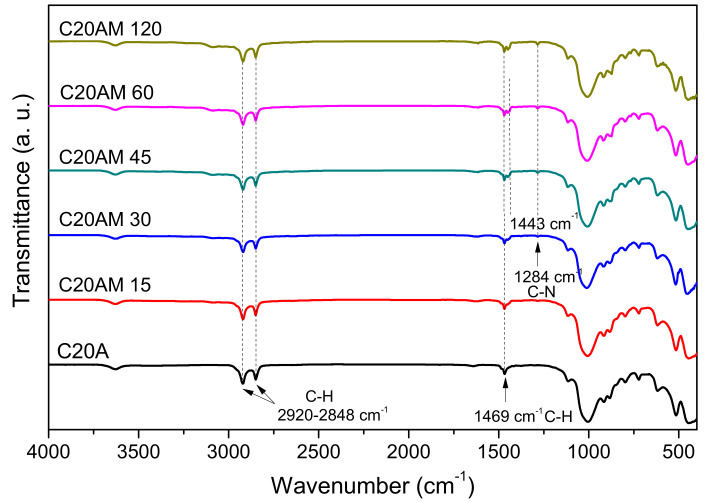
FT–IR spectra of the unmodified nanoclay C20A and the modified nanoclay C20A with 1,4–diaminobutane dihydrochloride.

The FTIR spectrum obtained from the unmodified and ultrasound-modified nanoclays at different times is shown in Figure 3. The signals located at 2920 cm^−1^ and 2848 cm^−1^ are attributed to the vinyl bonds C-H and C-H_2_, respectively [37]. A wide low signal at 3628 cm^−1^ corresponds to the O-H bond for the silicate and water [38]. The signal located at 1469 cm^−1^ corresponding to the C-H bond was identified in all samples. In the case of samples C20AM 45, C20AM 60, and C20AM 120, a second band is perceived at 1443 cm^−1^, this can be attributed to the bending of methylene, which indicates that the organic modifier has been exchanged in the galleries of the silicate coatings [39,40]. 

#### 3.1.3. X-ray Diffraction (XRD) 

**Figure 4:**
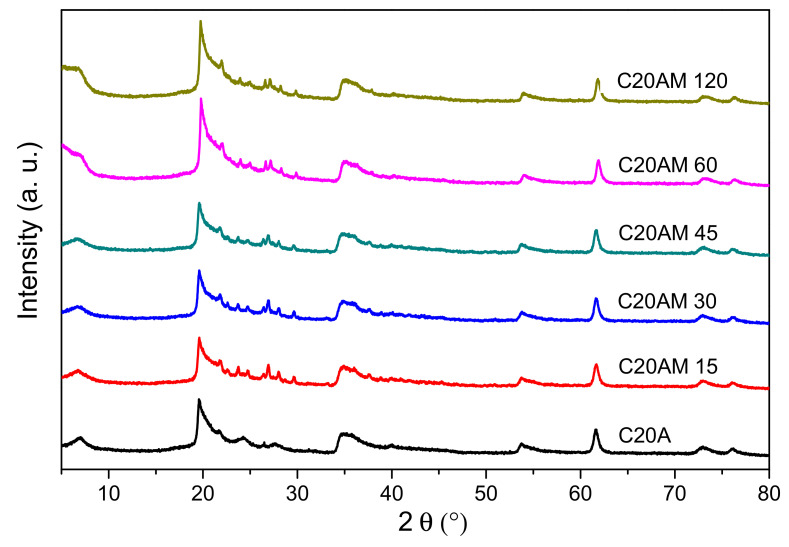
X-ray diffraction patterns of the unmodified nanoclay C20A and the modified nanoclay C20A with 1,4-diaminobutane dihydrochloride.

XRD patterns of the modified nanoclay with ultrasound and unmodified nanoclay are shown in Figure 4. All samples exhibit diffraction peaks at 61.72° and 35°. Additionally, a strong peak can be seen at 19.54°, characteristic of nanoclay C20A [41]. Modified nanoclays have small peaks between 20 and 30°, indicating that there is a change or chemical modification in their structure. Furthermore, samples C20AM 60 and C20AM 120 show signals at 10.05° and at 6.8°; in the literature it has been reported they that may present a prominent peak (d001 plane) at 2θ = 7.54°, corresponding to a basal spacing of 11.69 Å [42].

#### 3.1.4. Transmission Electron Microscopy (TEM)

**Figure 5:**
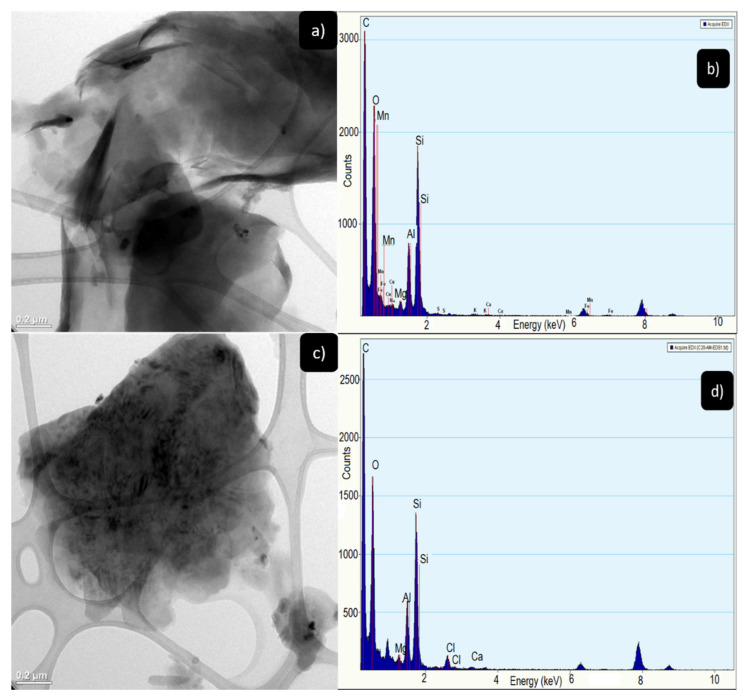
(**a**) TEM images closite 20A, (**b**) EDS spectra of the closite 20A, (**c**) TEM images C20AM 120 and (**d**) EDS spectra of the C20A 120.

In the images obtained by transmission electron microscopy (TEM), presented in Figure 5a, it is possible to observe the morphology of the C20A nanoclay, which is made up of agglomerated thin sheets with sizes smaller than 200 nm. In contrast, once the nanoclay is modified with the amine, it presents a different morphology as shown in Figure 5c, showing an agglomerate of sheets and the edge of the sheet is slightly thickened; this due to the presence of the amino groups on the surface of the nanoclay. 

Figure 5b shows the energy dispersive X-ray spectrum (EDS) of the cloisite 20A nanoclay, where the Si signal is found at 1.8 keV and the Al signal at 1.6 keV, characteristic elements of the nanoclay. Two signals corresponding to C and O. In the sample C20AM 120 the elements of Si, Al, C, O are presented and in 2.5 keV a signal related to Cl is shown; this due to the chemical modification that was carried out with 1,4-diaminobutane dihydrochloride.

### 3.2. Nanocomposites

#### 3.2.1. Fourier Transform Infrared Spectroscopy (FTIR) 

**Figure 6:**
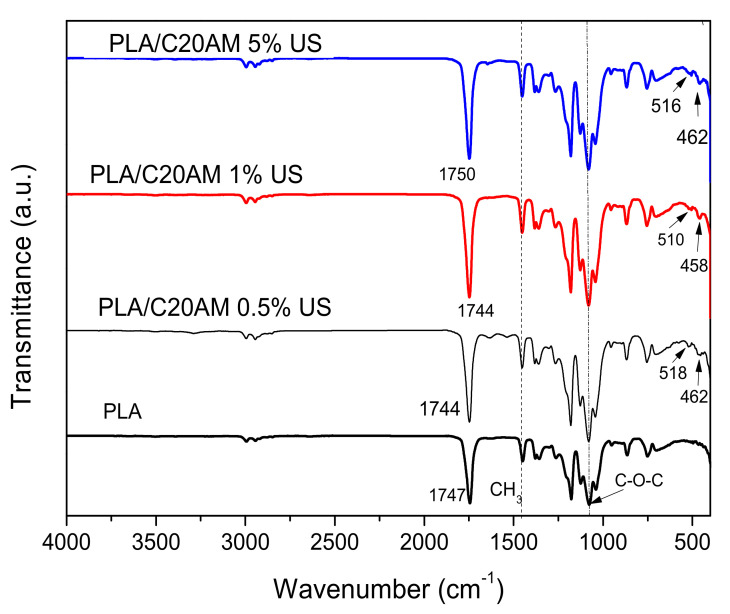
FT–IR spectra of nanocomposites with PLA and modified nanoclay at 0.5, 1, and 5% by weight.

FTIR spectra for the PLA and polymeric nanocomposites obtained with different concentrations of modified nanoclay during 120 min of ultrasonic treatment are shown in Figure 6. For the PLA resin and for the nanocomposites, signals are shown at approximately between 1744 and 1750 cm^−1^ corresponding to the carbonyl stretching, signals at values close to 1080 cm^−1^ corresponding to the C-O-C bonds can also be seen [43]. At a wavenumber of 1180 cm^−1^, an oscillating vibration of CH_3_ is presented and at 1389 and 1463 cm^−1^, signals corresponding to deformation vibrations of CH_3_ are shown [44]. For compounds containing nanoclay, modified signals are displayed at intervals of values from 458 to 618 cm^−1^, these are attributed to bending vibration Si-O-Si- and the A-O-Si-, respectively, related components montmorillonite [45]. This can be seen reflected in the samples with 0.5, 1, and 5% by weight of modified C20A nanoclay concentration, where these signals can be detected even when their loading percentage is low. The detection of Si, Al, and O from the concentration of 0.5% of C20A can be attributed to the excellent dispersion that was obtained thanks to the ultrasound-assisted extrusion technique, which achieves the homogeneous distribution of the nanoparticles in the polymer matrix [46]. 

#### 3.2.2. X-ray Diffraction (XRD) 

**Figure 7:**
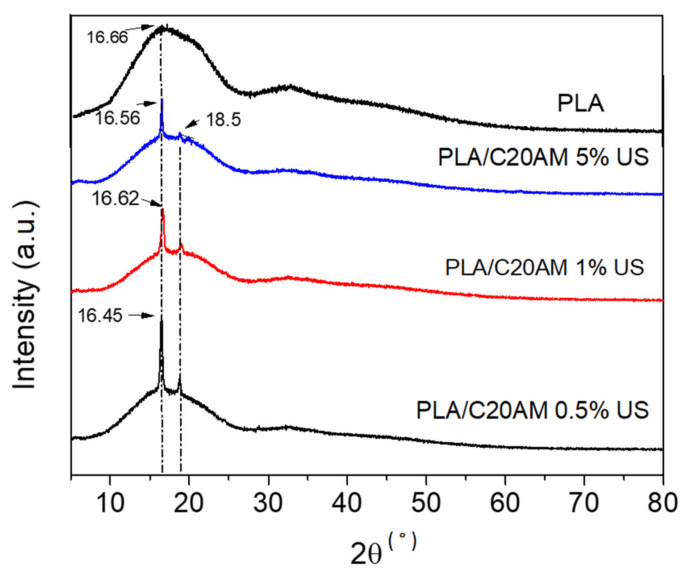
X–ray diffraction patterns of PLA and samples containing 0.5, 1, and 5% of C20A modified.

Figure 7 shows the diffraction patterns of PLA and nanocomposites with contents of 0.5, 1, and 5% of modified C20A. The main signal at 16.6° corresponds to a 5.9 Å interplanar spacing of the PLA [47]. For this polymer the pattern is characteristic, derived from the orthorhombic form containing 20 units in the crystalline cell [48]. In PLA and the three nanocomposites that were manufactured, they present a signal at 16° of the 2θ angle, particularly in nanocomposites.

In addition, in nanocomposites, a signal associated with the crystals of the PLA homopolymer becomes visible at 18.5°, as reported by Sajjad Saeidlou et al. in 2012, who studied the formation of stereocomplexes between poly (L-lactic acid) and poly (D-lactic acid) in the molten state [49]. These signals at 16 and 18.5° that protrude in the nanocomposites can be attributed to an increase in the crystallinity of the PLA polymer due to the presence of galleries of the modified nanoclay in low proportion, and because there is a better dispersion of the clay within the polymeric matrix acting as a good nucleating agent [50].

#### 3.2.3. X-ray Photoelectron Spectroscopy

**Figure 8:**
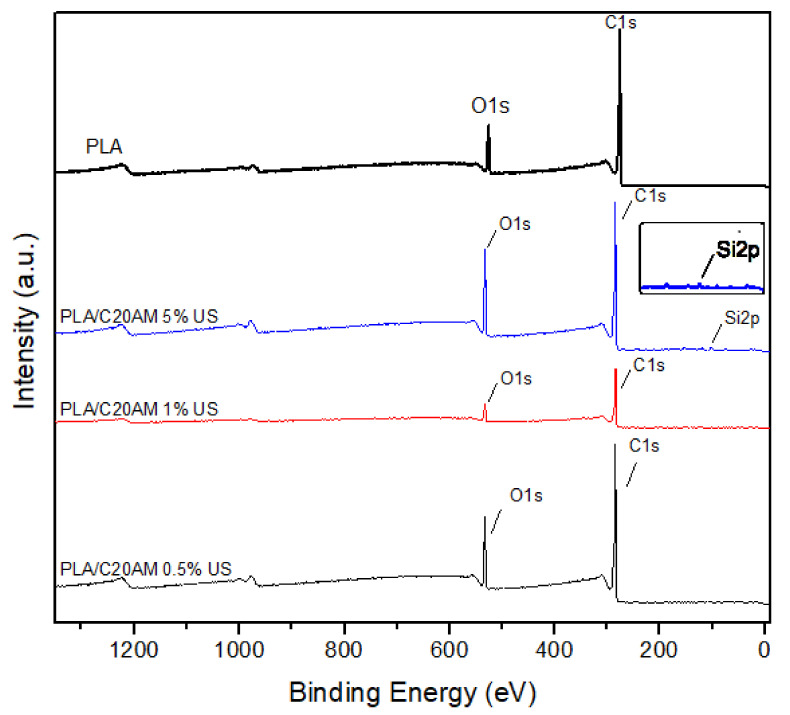
XPS spectra of PLA and nanocomposites were obtained.

To corroborate the elements present in the nanocomposites and their atomic percentages, XPS analyzes of the PLA polymer and nanocomposites were performed (Figure 8). For PLA resin, signals corresponding to C1s (284.73 eV) and O1s (533.16 eV), derived from the composition of said polymer, were observed. For the compounds of PLA/C20AM 0.5% US and PLA/C20AM 1% US, C1s and O1s signals were presented in the same way as PLA. In the data of atomic percentages provided by the team (Table 4), they indicated that these samples contained low percentages of Si2p 0.6 and 1.51%, respectively, but in the XPS spectrum the signal was very weak and could not be appreciated. For the PLA/C20AM 5% US sample, signals related to the Si2p present in the nanocomposite could be perceived at a binding energy of 102.19 eV. The signals obtained in the XPS analyses agree with those obtained by Frey et al. 2007; when evaluating PLA fibers with biotin using XPS, they observed that there was a streptavidin bond between PLA and biotin [51].

#### 3.2.4. Thermogravimetric Analysis (TGA)

**Figure 9:**
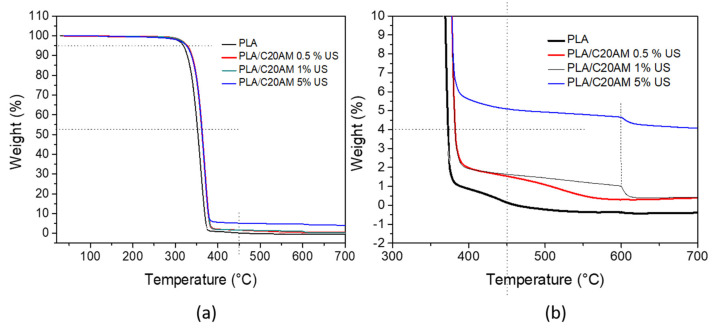
(**a**) Thermogravimetric analysis of the PLA and the nanocomposites obtained and (**b**) close-up of the thermogram obtained from 300 to 700 °C.

The TGA analysis was carried out in order to evaluate the different weight losses with respect to temperature and to consider the behavior of the organic and inorganic materials present in the nanocomposite. The results of the thermogravimetric analyses are shown in Figure 9a. For all samples including PLA alone, the first weight loss occurred from 312 to 389 °C. In Figure 9b an approach from 300 to 700 °C of the TGA thermogram of the nanocomposites is presented, where it can be observed that at 599 °C there is a small weight loss for the samples that contain 1 and 5% by weight of modified nanoclay. This behavior can also be observed in the TGA of the modified nanoclay (Figure 2), related to the release of olefin and amine because this nanoclay was previously modified with this type of organic group.

In addition, it can be observed that as the clay content increases, the thermal stability increases with respect to PLA pristine, because clay, as well as different ceramic materials, when interacting with the polymeric matrix, can form strong interactions between oxygen or hydrogen present in the nanoclay and the polymer chains or segments; this interaction causes greater thermal stability of the polymer chains. This performance has also been reported by Darie et al. in 2018, indicating a barrier effect of the clay toward polymer decomposition ablation products [52]. 

#### 3.2.5. Scanning Electron Microscopy of Nanocomposites (SEM)

**Figure 10:**
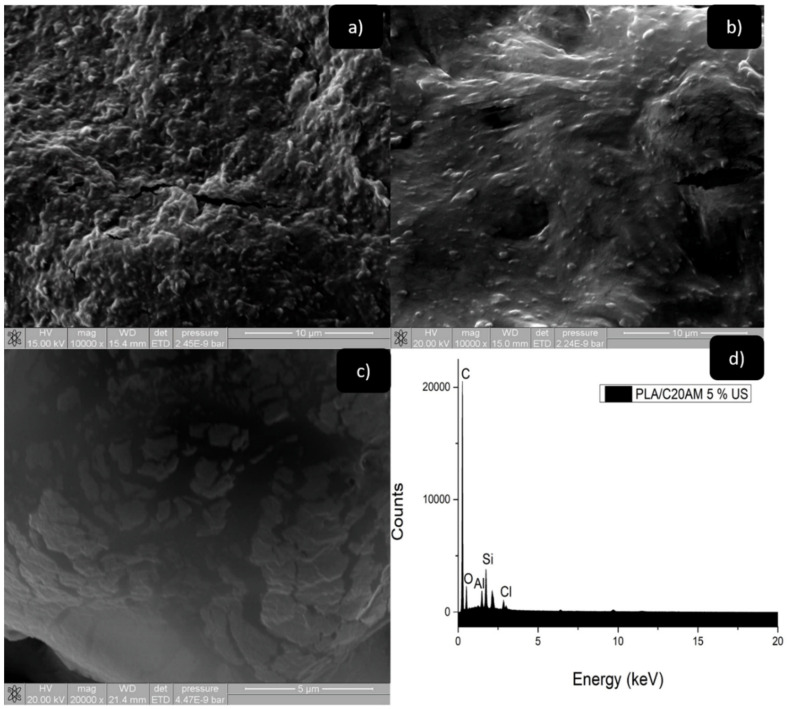
SEM images of nanocomposite (**a**) PLA/C20AM 1% at 10,000×, (**b**) PLA/C20AM 5% at 10,000×, (**c**) PLA/C20AM 5% at 20,000× and (**d**) EDS PLA/C20AM 5%.

The morphology of polymer nanocomposites was analyzed by scanning electron microscopy (SEM) to verify the degree of dispersion, and the arrangement of the clay sheets in the polymer matrix. The micrographs of the nanocomposites PLA/C20AM 1% and PLA/C20AM 5% at 10,000× (Figure 10a,b, respectively), showed good dispersion and apparently an interfacial adhesion of the nanoparticles in the polymeric matrix. This was mainly due to the ultrasound-assisted extrusion method used and to the modification of the nanoclay resulting in good compatibility of the nanoclay and the polymeric matrix. Mohapatra et al. reported a morphology similar to that of this study for PLA/PBAT/layered silicate blend nanocomposites [53].

Figure 10c shows the micrograph for the PLA/C20AM US sample at 5% at 20,000×. As can be seen in this figure, there is a better degree of dispersion and intercalation when implementing the modified clay [54]. Although agglomerates are seen, they are small and a lower number of tactoids is observed in this sample and a good degree of dispersion and intercalation [55]. The spectrum of energy dispersive X-ray spectroscopy (EDS) is shown in Figure 10d, where the presence of the elements of C and O, belonging to the polymeric matrix, in addition to Si and Al due to the incorporation of the nanoclay as reinforcement as well as the Cl element present in the amine with which the nanoclay was modified. These results agree with those obtained in X-ray diffraction, where a greater displacement towards low angles was obtained, even with high contents of 5% of C20AM clay. This behavior was attributed to the difference in the surface structure of the clay, which contributes to generating steric impediments for the free penetration of the polymer chains into their galleries.

### 3.3. Adsorption of Uremic Toxins and Dyes

#### 3.3.1. Percentage of Toxin Removal as a Function of Time

**Figure 11:**
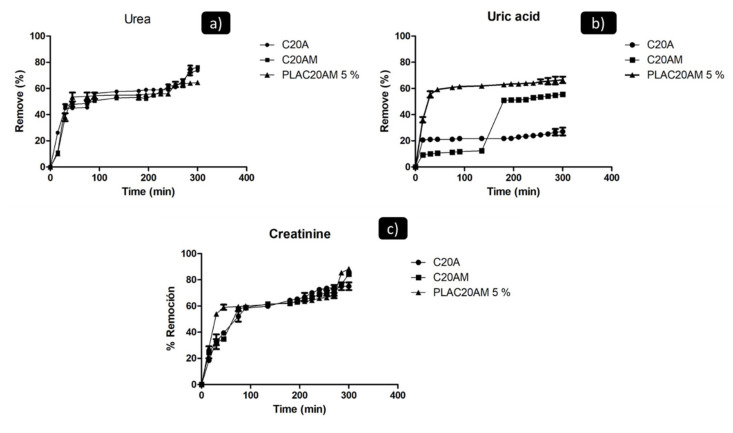
Removal percentage of (**a**) urea, (**b**) uric acid and (**c**) creatinine of the C20A, C20AM and PLAC20AM 5%. (Urea, uric acid and creatinine concentration = 160 mg/L, nanoclay and nanocomposite 20 mg/20 mL and t = 300 min).

To evaluate the adsorption of toxins and the effect of the modification carried out on the C20A clay and the PLA/C20AM-5% US nanocomposite, the percentage of removal of urea, creatinine, and uric acid were calculated using a calibration curve under the conditions described in the experimental section. The analysis was carried out over 4 h, sampling every 15 min. Figure 11a shows the percentage adsorption isotherm for urea. The amine-modified nanoclay showed good urea adsorption capacity with a removal percentage of 77%, while the unmodified C20A clay has 74%, this can be attributed to a better interaction between urea and nitrogen atoms present on the surface of the C20A. In the case of the nanocomposite, a urea removal percentage of 65% was obtained, which indicates that there is a good dispersion of the nanoclay in the polymeric matrix of PLA.

The uric acid adsorption percentage isotherm is presented in Figure 11b, the samples show values ranging from 25% to 70% removal. Some factors that can affect the adsorption of uric acid in materials are temperature, concentration, and Ph [56]. The PLAC20AM 5% nanocomposite obtained an elimination percentage of 70%, other reported values for the removal of uric acid with Nylon 6 and carbon black polymer nanocomposite were of 78 and 65%, nevertheless, the advantage of this PLA/nanoclay polymer nanocomposite is that it is biodegradable and biocompatible for a possible application in the area of health or environment [24,57].

Figure 11c shows the creatinine adsorption percentage isotherm, starting at 240 min. 75% removal is obtained for the unmodified nanoclay, whereas for the modified nanoclay and the nanocomposite there are values of 84 and 88% removal, this agrees with the results of Ramos et al. who tested modified zeolite for toxin adsorption [58].

Table 5 shows the results of the Langmuir and Freundlich adsorption parameters, comparing the correlation coefficients (R_2_). The values are higher for the Langmuir isotherm in the case of uric acid and creatinine, which suggests adsorption on the monolayer surface [25]. With urea, the Freundlich isotherm was adjusted for the nanoclay samples, this type of isotherm describes a multilayer adsorption process for heterogeneous surfaces [59].

#### 3.3.2. Percentage of Adsorption Efficiency of Methylene Blue (MB) as a Function of Time

**Figure 12:**
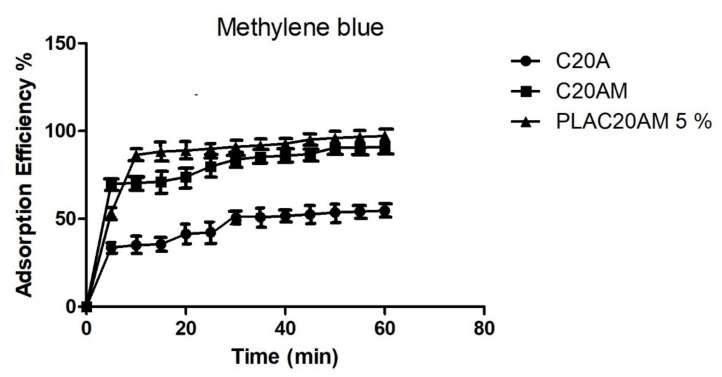
The adsorption efficiency of C20A, C20AM and PLAC20AM 5% (MB concentration = 200 mg/L, nanocomposite content = 20 mg/20 mL, T = 25 °C and 60 min).

The data of the percentage of adsorption efficiency of methylene blue as a function of time are shown in Figure 12. The C20A clay exhibits behavior in which the adsorption increases with time, after 30 min the adsorption stabilizes to reach a maximum adsorption efficiency of 55% at 60 min. On the other hand, clay modified with amino groups has a 91% adsorption efficiency, which is reached after 50 min. This may be due to the chemical interaction of amino groups with methylene blue, which allows excellent adsorption. The PLA-based biocomposite and the modified clay presented an adsorption efficiency of 97%. Mascarenhas et al. prepared by electrospinning technique faujasite zeolite (FAU)/poly (lactic acid) (PLA) composite fibers and managed to obtain an adsorption efficiency of 90% of methylene blue in 90 min [60]. In this study, adsorption was achieved in less time (60 min). Because of this, the use of this biocomposite as a cationic dye adsorbent is promising. 

Langmuir and Freundlich isotherm models were studied for the adsorption of methylene blue in an aqueous solution, the results are presented in Table 6, where it is observed that the clay and the nanocomposite conform to the Langmuir model, which suggests that they exist in homogeneous one-layer type adsorption sites. Vanaamudan et al. studied the adsorption of methylene blue on chitosan and Cloisite 30B nanocomposite, which was also adjusted to the Langmuir model [61].

#### 3.3.3. Effect of pH on the Adsorption MB

**Figure 13:**
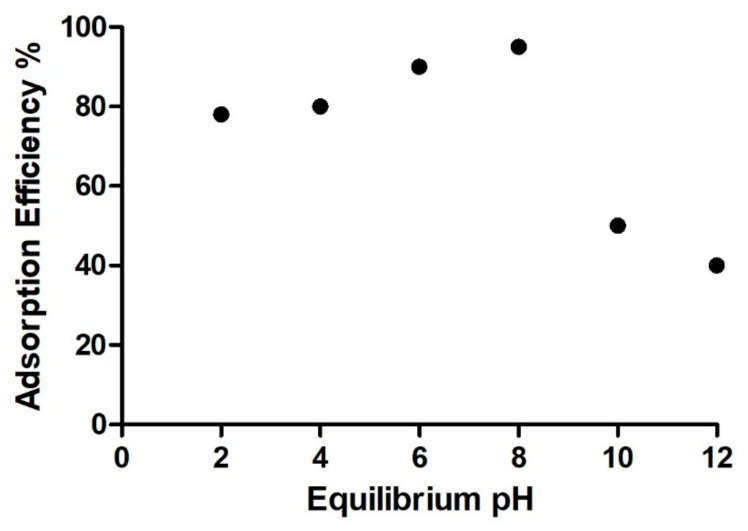
Adsorption efficiency % of MB a different pH (MB concentration = 200 mg/L, nanocomposite content = 20 mg/20 mL, T = 25 °C and 60 min).

The percentage of adsorption efficiency of MB at different pH is shown in Figure 13. Most of the dyes have different charges, some are anionic and others cationic when dissociated in an aqueous solution; the degree of adsorption is affected by this behavior due to the surface charge and pH of the solution [62,63]. The pH of the solution influences both the aqueous chemistry and the binding sites on the surface of the adsorbents, in this case, the maximum adsorption efficiency was at pH = 8 with 97%. On the other hand, at basic pH, the adsorption efficiency decreases. This may be due to the fact that the biocomposite acquires positive charges and as methylene blue has a cationic character, electrostatic repulsion occurs, which does not allow efficient adsorption on the biocomposite. This behavior has been previously reported for mineral clay materials [64].

#### 3.3.4. Comparative Adsorption Studies 

Table 7 shows the removal percentages for uric acid, creatinine, urea and methylene blue of various materials among which are ceramic nanoparticles, carbonaceous materials, polymers and those that were studied. According to Li et al. the adsorption has been considered as an attractive method to remove uremic toxins and methylene blue from aqueous solutions due to its low cost, and ease of operation. Some characteristics of adsorbent materials are large specific surface area, low density, chemical stability, suitability for large scale production, variety of structural forms, and the ability to modify the pore structures [65]. This study shows superiority in creatinine and methylene blue adsorption compared to the other listed materials.

#### 3.3.5. Desorption Studies

**Figure 14:**
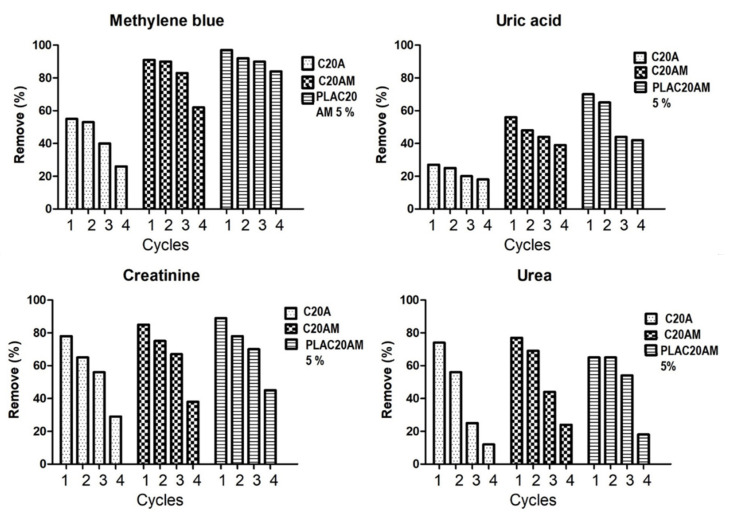
Effect of desorption cycles on methylene blue removal (MB concentration = 200 mg/L, nanoclay and nanocomposite content = 20 mg/100 mL, T = 25 °C and 60 min) or uremic toxins (uric acid, urea, creatinine concentration = 160 mg/L, nanoclay and nanocomposite 20 mg/100 mL and t = 60 min).

Adsorption-desorption cycles of the nanoclay and nanocomposite were determined and the percentage remove of each cycle was calculated as shown in Figure 14. In the case of uremic toxins, the removal percentage decreased with the number of cycles. This could be ascribed to the incomplete desorption of uremic toxins adsorbed on the nanoclay and nanocomposite. From the third cycle, a significant decrease in the removal percentage is observed. On the other hand, methylene blue presents a slight decrease in the adsorption capacity, for example, the percentage removal of 97 of the PLAC20AM 5% after four cycles is 90%.

## 4. Conclusions

In this study, C20A nanoclay/PLA was fabricated and (cloisite 20A) was modified with 1,4-diaminobutane dihydrochloride and tested for removal of uremic toxins and methylene blue dye. TGA, DRX, FTIR and TEM-EDS showed that a change in the results of the analyses occurred in the samples modified with 1,4-diaminobutane dihydrochloride and the best treatment time was 120 min of sonication.

Nanocomposites with PLA and modified clay were obtained. These materials showed in the morphological and physicochemical evaluations that the clay in low concentration was well dispersed due to the ultrasound-assisted extrusion method. The best removal percentage of uremic toxins and methylene blue was achieved with the PLA/C20AM 5% nanocomposite. The adsorption time of methylene blue was quantified in 60 min. One aspect that should be highlighted is that the amino group and the structure of the nanoclays in the nanocomposite obtained contributed significantly to the absorption of uremic toxins and methylene blue dye.

## Figures and Tables

**Table 1:** Identification of the samples according to treatment time.

Sample	Time of Sonication (min)	Amine Employed
C20A	0	None
C20AM 15	15	1,4–DHCDB
C20AM 30	30	1,4–DHCDB
C20AM 45	45	1,4–DHCDB
C20AM 60	60	1,4–DHCDB
C20AM 120	120	1,4–DHCDB

**Table 2:** Compounding formulations of poly (lactic acid)/cloisite 20A prepared by ultrasound-assisted melt extrusion process.

Sample Identification	Weight Percent of Additive (%)	Nanoarcilla C20A (g)	PLA Content (g)	Total Weight (g)
**PLA**	0	0	200	200
**PLA/C20AM 0.5% US**	0.5	1	199	200
**PLA/C20AM 1% US**	1	2	198	200
**PLA/C20AM 5% US**	5	10	190	200

**Table 3:** Thermal properties of C20A nanoclay modified at different times.

Sample	T_5%_ (°C)	T_25%_ (°C)
C20A	264.0	367.4
C20AM 15	230.9	355.9
C20AM 30	227.6	348.9
C20AM 45	226.9	339.3
C20AM 60	226.0	326.6
C20AM 120	226.5	307.4

**Table 4:** XPS data in terms of binding energy and atomic percentage of the samples obtained.

Sample	C1s Peak (eV)	O1s Peak (eV)	Si2p Peak (eV)	C1s At%	O1s At%	Si2p At%
**PLA**	284.73	533.16	ND	87.32	12.68	ND
**PLA/C20AM 0.5% US**	284.79	533.24	102.15	81.86	17.54	0.6
**PLA/C20AM 1% US**	284.74	533.06	102.17	80.82	17.67	1.51
**PLA/C20AM 5% US**	284.75	532.94	102.19	78.87	16.46	4.67

**Table 5:** Parameters of the isotherm constants and correlation coefficients for the adsorption urea, uric acid and creatinine.

Sample	Langmuir			Freundlich		
	k	q_max_	R^2^	n	K_F_	R^2^
**Urea**						
C20A	0.11	1.17	0.9137	0.25	0.58	0.7834
C20AM	0.10	0.73	0.9558	0.32	0.58	0.7514
PLAC20AM 5%	0.41	11.94	0.7444	0.09	1.8	0.9049
**Uric acid**						
C20A	0.44	22.82	0.8616	0.21	0.78	0.7100
C20AM	0.58	68.97	0.9991	0.05	1.41	0.9976
PLAC20AM 5%	0.12	44.78	0.9941	0.69	9.86	0.9185
**Creatinine**						
C20A	0.28	7.09	0.8652	0.99	6.06	0.8890
C20AM	0.20	412	0.9377	1.17	6.48	0.8656
PLAC20AM 5%	0.25	634	0.9721	1.06	6.56	0.9609

**Table 6:** Parameters of the isotherm constants and correlation coefficients were calculated for MB adsorption.

Methylene Blue						
Sample	Langmuir			Freundlich		
	k	q_max_	R^2^	n	K_F_	R^2^
C20A	0.04	266	0.976	1.10	8.98	0.9728
C20AM	0.015	788	0.9973	0.20	5.11	0.9736
PLAC20AM 5%	0.03	367	0.9864	0.16	4.91	0.7232

**Table 7:** Removal percentages of uremic toxins and dyes (adsorption, %).

Material	Uremic Toxins (Adsorption, %)			Dyes (Adsorption, %)	
	Uric Acid	Creatinine	Urea	Methylene Blue	References
ZnFe_2_O_4_	21	77	84		[59]
Zn_0·5_ Mg_0·5_ Fe_2_O_4_	20	77	85		[59]
Zno/Activated carbon	90				[66]
MCB/Nylon 6	78–82				[29]
ZnO/Nylon 6				93	[2]
CdS/PLA				90	[67]
Chitosan/sMMt				~94	[68]
C20A	27	78	74	55	Present study
C20AM	56	85	77	91	Present study
PLAC20AM 5%	70	89	65	97	Present study

## Data Availability

The data presented in this study are available on request from the corresponding author.
